# CFTR-driven immune microenvironment reprogramming synergizes with anti-PD-L1 antibody in hepatocellular carcinoma

**DOI:** 10.1038/s41419-026-08907-7

**Published:** 2026-06-10

**Authors:** Yijia Xu, Jianfang Sun, Chongyang He, Yipin Xie, Yuying Lu, Yujie Fan, Liying Yang, Mengying Zhang, Xin Liu, Lijuan Kong, Yanfeng Liu, Jinghai Zhang, Yang Su, Mingyi Zhao

**Affiliations:** 1 School of Life Science and Biopharmaceutical Sciences, Shenyang Pharmaceutical University, Shenyang, China; 2 Department of Neurosurgery, Shengjing, Hospital of China Medical University, Shenyang, China; 3 Department of General Surgery, Shengjing Hospital of China Medical University, Shenyang, China

**Keywords:** Cancer microenvironment, Tumour biomarkers

## Abstract

Immune checkpoint inhibitors, notably PD-1/PD-L1 monoclonal antibodies, are now used as first-line treatments for hepatocellular carcinoma (HCC) and have improved outcomes for some patients. Nevertheless, their response rate remains below 30%, largely due to HCC’s immunosuppressive microenvironment and insufficient T-cell infiltration. Strategies to address these limitations are urgently needed. Ion homeostasis has been recognized as a pivotal factor in modulating the tumor immune microenvironment. This study found that the expression and functionality of CFTR, a chloride channel, in human HCC tissues and cells were negatively correlated with HCC progression. Furthermore, it was utilized human/murine HCC cell lines, mouse models, and human HCC organoids to investigate how CFTR activation (genetic/pharmacological) impacts HCC biology. Key assessments included: (1) tumor cell ion homeostasis, (2) downstream signaling proteins, (3) tumor microenvironment cytokine levels, and (4) functional changes in TAMs, CD8 + T cells, and Tregs. We further assessed combined effects with PD-L1 antibodies to explore CFTR-immune checkpoint interplay, providing multidimensional insights into CFTR-mediated HCC pathogenesis and immunomodulation. The results showed that upregulation of CFTR expression and channel activity reduced intracellular Cl⁻ and Ca²⁺ concentrations in HCC cells, thereby suppressing the expression of chloride-associated transcription factor RUNX1 and calcium pathway transcription factor NF-κB. This regulatory mechanism orchestrates the synthesis and secretion of cytokines/chemokines (CSF-1, TGF-β, CCL20, CCL22), driving macrophage polarization toward M1 phenotype while promoting M1 macrophage and CD8 + T lymphocyte infiltration. Concurrently, it suppressed Treg proliferation and tumor infiltration. These immunomodulatory effects synergistically enhanced PD-1/PD-L1 immune checkpoint blockade efficacy, ultimately augmenting therapeutic outcomes in HCC. Therefore, this study establishes CFTR potentiation as a novel immunomodulatory axis, proposing biomarker-driven combination regimens to enhance therapeutic efficacy while mitigating immune-related adverse events, thereby addressing an urgent unmet need in translational hepatology.

**Figure:**
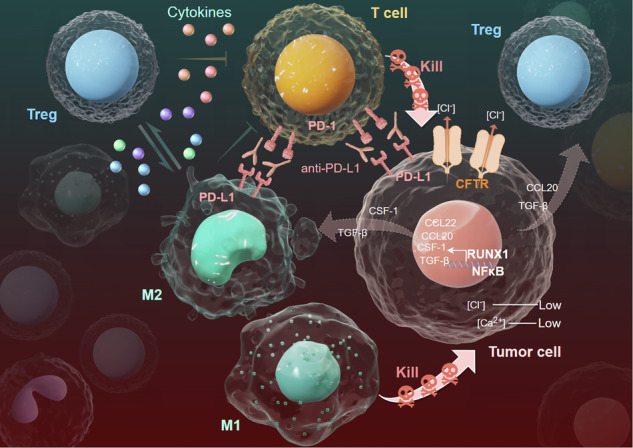

## Introduction

Hepatocellular carcinoma (HCC) stands as one of the most prevalent malignant cancers worldwide, exhibiting persistently elevated incidence and mortality rates. Recent advancements in the investigation of tumor immune microenvironments have progressively unveiled the distinctive immunological characteristics of HCC. Analogous to other malignancies, the HCC immune microenvironment constitutes a sophisticated ecosystem comprising diverse immune and non-immune cellular components. Notably, HCC demonstrates unique immunocyte composition and proportional distribution patterns, which predominantly emerge within chronic inflammatory contexts. This pathologically distinct microenvironment is postulated to be a primary driver of the tumor’s exceptional immune cell infiltration profile, potentially accounting for its distinctive immunobiological behavior and therapeutic resistance mechanisms [1].

The quantitative and phenotypic characteristics of tumor-infiltrating lymphocytes within the HCC microenvironment exhibit significant prognostic correlations [2, 3]. Notably, HCC demonstrates reduced infiltration densities of CD8+ cytotoxic T lymphocytes and natural killer (NK) cells, while displaying disproportionate enrichment of regulatory T cells (Tregs) and myeloid-derived suppressor cells (MDSCs). This pathological cellular imbalance perpetuates a chronically immunosuppressive microenvironment [4]. Comparative analyses reveal that HCC maintains substantially higher immunosuppressive cell ratios than other solid malignancies, including breast and colorectal carcinomas, with Treg populations accounting for up to 30% of total immune infiltrates in HCC lesions [4, 5].

Furthermore, HCC microenvironments are characterized by substantial infiltration of M2-polarized tumor-associated macrophages (TAMs). These effectors facilitate neoplastic progression through dual mechanisms: secretion of immunosuppressive cytokines and active promotion of tumor angiogenesis. Additionally, comparative immunohistochemical studies demonstrate significant quantitative and functional alterations in dendritic cell (DC) populations within HCC tissues relative to non-neoplastic hepatic counterparts. These DCs exhibit impaired antigen-presenting capacity and enhanced tolerogenic programming, collectively contributing to immune evasion mechanisms [1].

The synergistic interplay of these immunomodulatory components establishes a profoundly immunosuppressive niche in HCC. This pathologically remodeled microenvironment not only fosters primary tumor proliferation but also creates permissive conditions for metastatic dissemination through multiple mechanisms, including extracellular matrix remodeling and vascular niche formation.

The advent of immune checkpoint inhibitors (ICIs) has revolutionized oncotherapeutic paradigms in recent years, with emerging clinical evidence demonstrating substantial therapeutic potential in HCC management. Nivolumab, a pioneering PD-1 monoclonal antibody, received FDA accelerated approval in 2017 as second-line therapy for advanced HCC. Phase II clinical trial data involving 262 treatment-refractory HCC patients revealed an objective response rate (ORR) of 20% and a disease control rate (DCR) of 64% following nivolumab monotherapy. Subsequent phase III investigation (*n* = 743 systemic treatment-naive HCC patients) demonstrated nivolumab’s superiority over sorafenib in multiple clinical parameters, including durable disease control duration, improved safety profile [6].

This therapeutic breakthrough catalyzed the clinical development of multiple PD-1/PD-L1 inhibitors in HCC. Notably, the combination regimen of atezolizumab (anti-PD-L1) and bevacizumab (anti-VEGF) achieved landmark FDA approval in 2020 as a first-line indication for unresectable HCC, establishing new standards of care based on superior progression-free survival outcomes [7].

Despite these advancements, critical challenges persist in HCC immunotherapy: (1) Suboptimal response rates limited by tumor immunogenicity barriers; (2) Development of acquired resistance through compensatory immune checkpoints activation; (3) Immune-related adverse events (irAEs) affecting 25–30% of patients; (4) Imperative optimization of combination strategies targeting synergistic pathways to overcome therapeutic plateau [8]. Current translational research focuses on predictive biomarker development and spatial multi-omics characterization of immune exclusion phenotypes to refine patient stratification.

With the publication of the Nobel Prize in Physiology or Medicine in 2021, ion channel proteins expressed on cell membranes have attracted significant attention again as potential therapeutic targets for diseases. These channel proteins can be used as upstream factors to regulate the occurrence and development of diseases, and are potential targets for disease prediction and treatment with great clinical significance. Over recent years, the relationship between ion channel function and tumors has also attracted increasing attention. These channels appear to act as an upstream factor to regulate tumor proliferation and apoptosis, therefore participating in tumor angiogenesis, deterioration and metastasis [9–11].

Cystic fibrosis transmembrane transduction regulator (CFTR), one chloride channel protein composed of 1480 amino acids, plays important roles in the transepithelial transport of salts, the regulation of ion concentration and liquid flow [12, 13]. The occurrence and development of many diseases are closely related to the abnormal expression or function of CFTR, such as pulmonary fibrosis, chronic pancreatitis, and cancers [12, 14, 15]. Under physiological conditions, CFTR is a chloride channel that is widely distributed at the top of the intima in hepatocytes, bile ducts and gallbladder epithelial cells. This channel participates in bile secretion; dysfunction in this channel will lead to intrahepatic cholestasis.

In this study, CFTR was found to be expressed at significantly lower levels in liver cancer tissues when compared between cancerous tissues and adjacent tissues from liver cancer patients. The expression levels of CFTR in HCC were negatively correlated with clinical stage and positively correlated with the degree of differentiation. In order to explore the correlations and potential mechanisms between CFTR and HCC, this study firstly detected lower expression levels of CFTR in HCC tissues and HCC cells by database prediction, HCC cells and human HCC tissues samples, and found that the lower expression levels of CFTR were significantly correlated with the proliferation and metastasis of HCC. Emerging evidence underscores CFTR as a potential modulator of tumor immunogenicity, yet its precise role in shaping the immunosuppressive landscape of HCC remains mechanistically undefined. To systematically investigate CFTR-mediated immunoregulation, we engineered stable CFTR-manipulated HCC models through lentiviral transduction, establishing isogenic cell lines with either CFTR knockdown (HepG2-shCFTR) or overexpression (MHCC97H-CFTR/Hepa1-6-mCFTR). These cellular tools were subsequently deployed across five immunocompetent and immunodeficient murine models, including HepG2-shCFTR orthotopic xenografts in nude mice, MHCC97H-CFTR orthotopic xenografts in nude mice, MHCC97H-CFTR orthotopic implants in NOD-SCID mice, Hepa1-6-mCFTR orthotopic tumors in C57BL/6 J mice, Hepa1-6-mCFTR subcutaneous tumors in C57BL/6 J mice, enabling comprehensive evaluation of CFTR’s impact on tumor progression and immune microenvironment remodeling. Mechanistic dissection integrated bioinformatics interrogation with multilayer experimental validation, encompassing Western blot analysis, qPCR quantification, and ELISA. Building upon these findings, we pioneered combinatorial therapeutic strategies employing PD-L1 blockade in CFTR-activated models, with parallel validation in patient-derived organoid platforms. Our findings establish CFTR potentiation as a novel immunomodulatory axis, proposing biomarker-driven combination regimens to enhance therapeutic efficacy while mitigating immune-related adverse events, thereby addressing an urgent unmet need in translational hepatology.

## Materials and methods

### Informatics approach

CFTR expression data in normal tissues and tumor tissues were downloaded from The Cancer Genome Atlas (TCGA) (https://gdc.cancer.gov/about-data/publications/pancanatlas), Gene Expression Profiling Interactive Analysis (GEPIA) (http://gepia.cancer-pku.cn/), and Genotype-Tissue Expression (GTEx) (https://www.gtexportal.org/home/) databases.

ESTIMATE, an algorithm in R-package “limma”, “estimate”, “ggplot2”, “ggpubr” and “ggExtra” was used to calculate stromalscore, immunescore, ESTIMATEScore and immunocytes infiltration.

Standardized pan-cancer data sets are downloaded from UCSC datasets, including TCGA, TARGET GTEx (PANCAN, N19131, C60499). The Timer method (version 0.99.9, https://www.ncbi.nlm.nih.gov/pmc/articles/PMC8283787/) of R-package IOBR was used to evaluate immune cells infiltration scores for each patient in each tumor based on gene expression. Expression data of marker genes of immune pathways (chemokine (41), receptor (18), MHC (21), Immunoinhibitor (24), Immunostimulator (46)) in each sample were extracted. The pearson correlations of CFTR and the marker genes of five immune pathways were calculated. Sixty immune checkpoint pathway genes (Initial (24), Stimulatory (36)) were extracted. Pearson correlation of CFTR and these marker genes was calculated. The “corr. test” command of R-package psych was used to calculate Pearson’s correlation coefficients of gene and immune infiltration scores in various tumors.

### Single-cell data analysis and cell cluster identification

The single-cell RNA sequencing (scRNA-seq) dataset GSE149614 was obtained from the GEO database (https://www.ncbi.nlm.nih.gov/), which includes single-cell transcriptomic data from ten HCC patients. Ten primary tumor samples and eight adjacent normal tissue samples were selected to explore differential gene expression at the single-cell level in HCC. Clusters identified through the UMAP analysis were manually annotated. Cluster annotation was based on a comparison of marker genes from the CellMarker 2.0 database (http://bio-bigdata.hrbmu.edu.cn/CellMarker/index.html).

### Spatial transcriptome data analysis

The spatial transcriptome data were derived from HRA000437, which included 12,672 spot and critical region slices of non-tumor liver, HCC, and adjacent Tumor, and Mendeley Data (skrx2fz79n), which included data of HCC cancer tissues from patients treated with anti-PD-1 therapy. The data were processed according to the default method of the R package Seurat.

### Human HCC tissues samples

A total of 48 patients with HCC were enrolled between June 2018 and July 2022 from Shengjing Hospital of China Medical University. Ethical approval was obtained from the Institutional Review Board of Shengjing Hospital (2022PS1101K).

### Cell culture and transfection

The HL7702, HepG2, MHCC97L, MHCC97H, AML12, Hepa1-6, THP-1, RAW264.7 cell lines were purchased from American Type Culture Collection (ATCC) and were grown in RPMI 1640/ DMEM medium containing 10% fetal bovine serum (FBS) and 1% penicillin-streptomycin (penicillin-streptomycin, Gibco, USA) at 37 °C in a 5% CO_2_ incubator. HBLV-hCFTRshRNA-ZsGreen-PURO, control plasmid HBLV-ZsGreen-PURO were purchased from Wanze Bio (Shenyang, China). hCFTR-GV492, hCFTR-CV146/mCFTR-CV146 and the control plasmid (Ctrl-GV492 and Ctrl-CV146) were purchased from Genechem (Shanghai, China). Monoclonal cell lines exhibiting stable interference/over-expression of CFTR were screened by puromycin and identified by Western Blot. Forskolin was obtained from MedChem Express (USA).

Primary Peripheral Blood Mononuclear Cells (PBMC, ATCC) were grown in RPMI 1640 medium (including 10% FBS and 1% penicillin-streptomycin) at 37 °C in a 5% CO_2_ incubator.

### Quantitative PCR analysis

Total RNA was isolated and reverse transcribed with a PrimeScriptTM RT Reagent Kit (Takara, Japan). PCR was performed with SYBR Premix Ex TaqTM (Takara, Japan) and a Mx3000P Real-Time PCR Detection System (Agilent). The following primers were used for the experiment: glyceraldehyde-3-phosphate dehydrogenase (GAPDH): forward: 5′-CCACTCCTCCACCTTTGAC-3′; reverse: 5′-ACCCTGTTGCTGTAGCCA-3′ CFTR: forward: 5′-CTTGCTCGTTGACCTCCACT-3′ and reverse: 5′-CAGAAGCGTCATCAAAGCAT -3′.

### Cell viability assays

In vitro cell viability was determined using the MTT assay. Cells (5 × 10^3^/well) were seeded in 96-well culture plates. The cells were then incubated for 24, 48, and 72 h at 37°C in a 5% CO_2_ incubator. Then, 5 mg/mL of MTT solution was added, followed by 100 μL of DMSO 4 hours later. Finally, absorbance was measured at 570 nm using a multi-mode plate reader (Molecular Devices, USA).

### Transwell assays

Cell invasion and migration were assessed using Transwell Permeable Supports (Corning, USA). Cells were resuspended in 100 μL of serum-free medium and plated onto Transwell filter inserts coated with Matrigel 1:8 (BD Biosciences, USA) for invasion assays, and without Matrigel for migration assays.

### Scratch-wound healing recovery assays

Cells seeded in 6-well cell culture plates were transfected at 60–70% confluency in triplicate. Growth medium was removed, and straight incisions were made with a standard 200 μL pipette tip. Cells were washed several times with PBS to remove detached cells.

### Western blotting

Total protein extraction was carried out using a Total Protein Extraction Kit (Kaiji, Nanjing, China). The concentration of extracted protein was then determined by a BCA Protein Assay Kit (Kaiji, Nanjing, China). Separated proteins were transferred to a PVDF membrane and then blocked with goat serum or 10% skimmed milk and then incubated with diluted primary antibodies (CFTR antibody: Santa Cruz, sc-376683, 1:1000; MMP2 antibody: Invitrogen, 436000, 1:1000; β-actin antibody: Invitrogen, PA1-183, 1:2000; GAPDH antibody: Invitrogen, 39-8600, 1:1000； CXCL9 antibody: Proteintech, 22355-1-AP, 1:500; CXCL10 antibody: Affbiotech, DF6417, 1:1000; CXCL11 antibody: Abcam, ab259863, 1:1000; Anti-NF-κB p65 antibody: STARTER, S0B0549, 1:1000; Goat anti-Rabbi antibody: Abcam, ab6721, 1:5000-1:20000; Goat anti-Mouse antibody: Abcam, ab6728, 1:5000-1:20000. β-actin and GAPDH were used as internal references. Each experiment was repeated at least three times to calculate mean values.

### Calcium (Ca^2+^) influx measurement

The cells of each group were collected and washed with Lock’s buffer (in mM: 5.6 KCl, 154 NaCl, 5.6 glucose, 1.0 MgCl2, 2.3 CaCl2, 0.1 glycine, 8.6 Hepes/NaOH, pH 7.4). Harvested cells were incubated for 1 h at 25 °C with dye loading buffer (5 × 10^6^ cells/mL) containing 10 μmol/L Fura2-AM. Then, cells were washed with Lock’s buffer and transferred to a black-bottomed 96-well plate. Cells were excited at 340 and 380 nm and detected at 505 nm by using a Tecan Infinite M200pro (TECAN, Swiss).

### Chloride ion (Cl^−^) influx measurement (MQAE)

Cells were inoculated in a 48-well plate, and when the fusion degree was moderate, the original culture medium was removed, 100 μL MQAE (MedChemExpress, USA) working solution was added to each well, incubated at 37 °C for 1 h, and then the orifice plate was washed with Krebs-HEPES buffer for 3 times. Five fields of each group were taken randomly under fluorescence microscope, and the fluorescence intensity was analyzed by Image J software, and the average fluorescence intensity was calculated.

### ELISA

For TGF-β, IL-10, CXCL12, CCL2 (human), CCL12 (mouse), CCL22, CSF-1, IL-4, IL-13 quantification, the ELISA Kits were used to measure the levels in tumor secretion.

### In vivo orthotopic HCC transplanted tumor models in BALB/c nude or C57BL/6 J or NOD-SCID mice

Human or mice HCC cells were injected into the liver of BALB/c nude, C57BL/6 J or NOD-SCID mice, and the wound was quickly sutured. One week later, PBMC-derived macrophages/T cells (2 × 10^6^ of each mouse) were injected into tumor-bearing NOD-SCID mice through the tail vein. Forskolin, PD-L1 antibody were injected into mice twice a week. Tumor growth and metastasis were observed in each group of mice once a week with a small animal in vivo imaging system (Bruker MI SE, Germany/ IVIS Lumina, PerkinElmer, USA). Four weeks later, the mice were sacrificed, and tumors in the liver and other tissues were removed, measured and weighed. Tumor tissues were stored in liquid nitrogen or formalin solution for subsequent analysis. This protocol was approved by the Committee on the Ethics of Animal Experiments of the Shenyang Pharmaceutical University (SYPU-IACUC-C2021-9-30-102, SYPU-IACUC-C2022-03-02-032) and Experiments of the Center for Model Biology Research of Saiye (TCM220719JX1).

### In vivo subcutaneous HCC transplanted tumor models in C57BL/6 J mice

Mice HCC cells were Subcutaneously injected into C57BL/6 J mice. One week later, Forskolin, PD-L1 antibody were injected into mice twice a week. Tumor growth and metastasis were observed in each group mice once a week with a small animal in vivo imaging system (IVIS Lumina, PerkinElmer, USA). 3 weeks later, the mice were sacrificed, and tumors in the liver and other tissues were removed, measured and weighed. Tumor tissues were stored in liquid nitrogen or formalin solution for subsequent analysis. This protocol was approved by the Committee on the Ethics of Animal Experiments of the Shenyang Pharmaceutical University (SYPU-IACUC-C2022-03-02-032).

### Multiplex immunohistochemical (mIHC) analysis

Tumor tissues were harvested and fixed in formalin at 4 °C for 24 h. After paraffin embedding, sections (5 μm) were dried and stained with antibody (CD8a antibody: Servicebio, GB12068, 1:1000; iNOS antibody: Abcam, ab15323, 1:1000; FoxP3 antibody: Servicebio, GB112325, 1:1000; HRP-goat anti-rabbit IgG: GB23303, 1:500) to visualize the protein status. Sections were photographed with an Olympus AX70 microscope.

### Human HCC organoids culture

Organoid culture medium was purchased from Absin Biologics. Fresh human HCC tissues were cut into small pieces, digested with collagenase, and processed into a single-cell suspension through cell screening. The cell suspension was evenly mixed with Matrigel (Corning, USA) under ice-cold conditions and dropped onto a 24-well culture plate. After 72 hours, the morphology of organoids was preliminarily diagnosed, and different concentrations of drugs were given. The growth status of organoids was observed under a microscope for 72 hours.

The cell suspension was also mixed with Matrigel under ice-cold conditions and dropped onto a 96-well culture plate. After 72 hours, the morphology of organoids was preliminarily diagnosed, and different concentrations of drugs were given. Organoid ATP Viability Assay Kit (Absin, China) was used to test organoid activity.

### Flow cytometry analysis

Tumor cells were minced into small pieces, and red blood cells were lysed with Pharmlyse buffer (BD Bioscience, CA, USA). The single cells were then passed through a 40-μm nylon mesh strainer and stained with the following antibodies to observe M1or M2 macrophages, CD8 + T cells and Treg cells: anti-CD11b-FITC (BD bioscience), anti-F4/80-APC (BD bioscience), anti-CD86-PerCP/Cyanine5.5 (Biolegend), anti-CD206-PE (Biolegend), anti-CD45-FITC (BD bioscience), anti-CD3e-APC (BD bioscience), anti-CD8a-PE (BD bioscience), anti-CD4-FITC (BD bioscience), anti-CD25-APC (BD bioscience), anti-Foxp3-PE (BD bioscience), CD11b-FITC (Biolegend), CD86-APC (BD bioscience), CD206-PE (BD bioscience), CD3e-FITC (BD bioscience), CD8a-PE (BD bioscience), CD4-FITC (BD bioscience), CD25-APC (BD bioscience), Foxp3-PE (BD bioscience). Cells were analyzed by FACS using flow cytometers (BD Biosciences, USA; ACEA Bioscience, USA) and analyzed by FlowJo and Novo Express software.

### Data analyses

Statistical analyses of the data are provided as mean ± SEM. Statistical significance is assessed by one-way ANOVA followed by Turdey’s post hoc test, *P < 0.05 and **P < 0.01.

## Results

### CFTR is expressed at low levels in HCC tissues and HCC cells and is correlated with progression

**Fig. 1:**
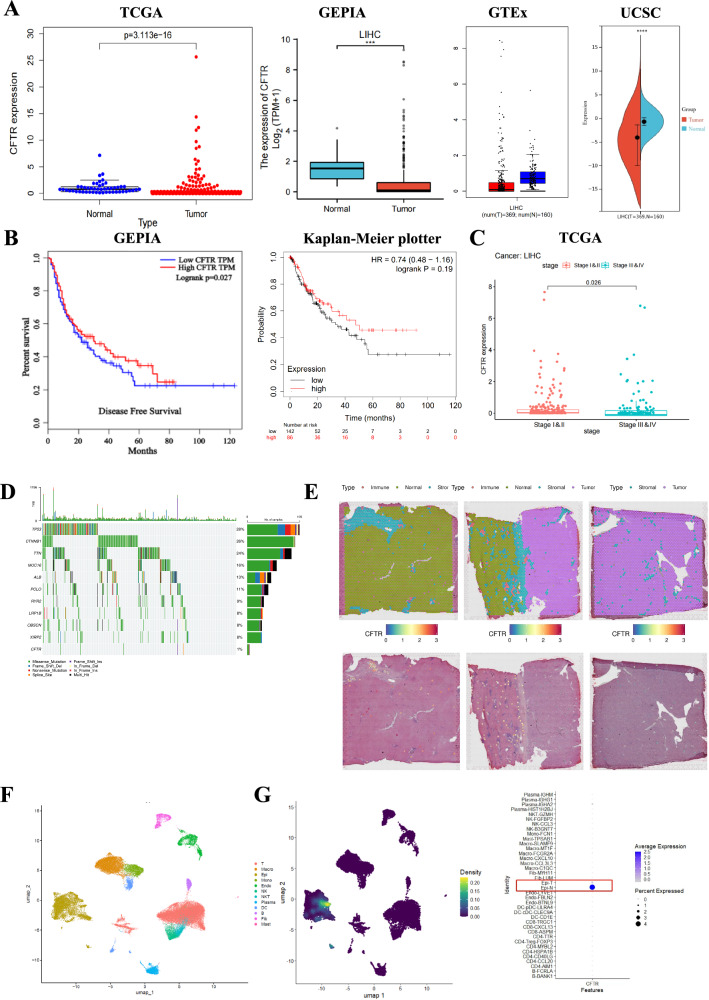
Multi-omics predictive results related to CFTR expression in hepatocellular carcinoma. **A** The predicted expression of CFTR through databases. **B** The predicted correlation of CFTR expression with prognosis. **C** The predicted correlation of CFTR expression with metastasis through TCGA. **D** Genomic characterization demonstrated a low somatic mutation frequency (1.35%, 5/370 cases) in the HCC cohort. **E** The representative spatial transcriptomics results of CFTR in HCC. **F** Single-cell clustering analysis was performed using the GSE149614 dataset. **G** CFTR expression was exclusively detected in non-malignant and malignant epithelial cells, whereas it was virtually undetectable in immune cells and stromal cells. Data are expressed as mean ± SEM. Statistical significance was concluded at *P < 0.05, **P < 0.01.

**Fig. 2:**
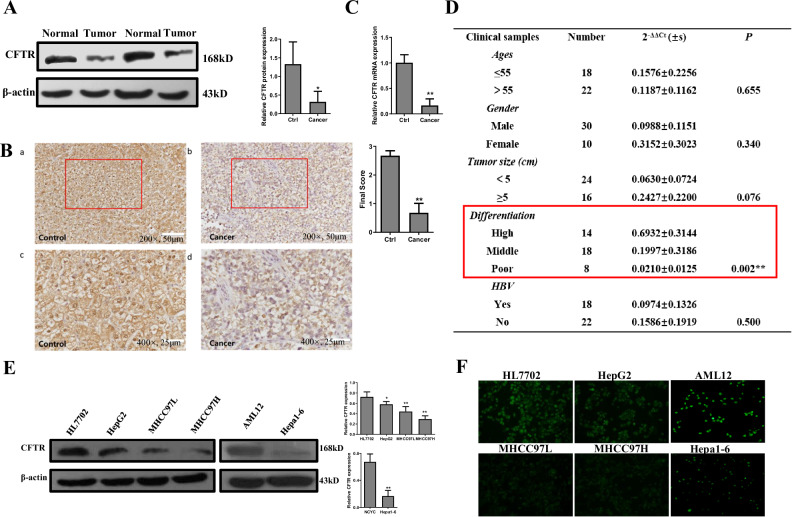
CFTR is lower expressed in human HCC tissues and HCC cells. **A** The representative western blot and statistical analysis of CFTR protein expression in patients HCC tissues (*n* = 8). **B** qPCR analysis of CFTR mRNA in patients HCC tissues (*n* = 20). **C** Immunohistochemistry analysis of CFTR in patients HCC tissues (*n* = 8). **D** The correlation between CFTR mRNA expression and differentiation in patients HCC tissues (*n* = 20). **E** CFTR expression in normal liver cells and several HCC cells (*n* = 3). **F** The influence of CFTR on intracellular chloride concentration by using MQAE (*n* = 3). Data are expressed as mean ± SEM. Statistical significance was concluded at *P < 0.05, **P < 0.01.

To systematically investigate the transcriptional and translational profiles of CFTR in HCC, we conducted comprehensive bioinformatics analyses utilizing multi-omics databases including The Cancer Genome Atlas (TCGA), Gene Expression Profiling Interactive Analysis (GEPIA), Genotype-Tissue Expression (GTEx), and UCSC Xena resources. Integrated bioinformatic analyses revealed significant downregulation of CFTR expression in HCC specimens compared to adjacent non-tumor tissues (Fig. 1A), with notable correlations between reduced CFTR levels and both diminished survival outcomes and advanced tumor staging (Fig. 1B, C). Genomic characterization demonstrated a low somatic mutation frequency (1.35%, 5/370 cases) in the HCC cohort (Fig. 1D), suggesting that transcriptional suppression rather than genetic alteration primarily drives CFTR dysregulation in hepatocarcinogenesis. Meanwhile, spatial transcriptomics results further demonstrated that in actual pathological sections of liver cancer, CFTR exhibited significantly lower expression levels in HCC tissues compared to adjacent non-tumor tissues (Fig.1E). To preliminarily elucidate the mechanism by which CFTR regulates tumor progression, a bioinformatics approach was employed to initially determine the expression profile of CFTR in HCC tissues. The single-cell RNA sequencing dataset GSE149614—comprising 10 primary HCC tissues and 8 adjacent normal tissues—was obtained from the GEO database. Cell population annotation was performed using the CellMarker 2.0 database (Fig. 1F). Based on this annotation, a systematic characterization of CFTR expression across distinct cell subpopulations was conducted (Fig. 1G). The results revealed that CFTR was exclusively detected in both non-malignant and malignant epithelial cells, but was nearly absent in immune and stromal cells. Thus, we hypothesize that CFTR may remodel the immune microenvironment by modulating specific signaling pathways within tumor cells. Validation experiments using clinical specimens showed concordant reduction of both CFTR mRNA and protein expression levels in HCC tissues compared to matched normal controls (Fig. 2A–C), and that the expression levels of CFTR were positively correlated with the prognosis and stage of HCC differentiation (Fig. 2D).

Then, we selected HL7702 human normal hepatocytes cells, AML12 mice normal hepatocytes cells and HepG2, MHCC97L, MHCC97H, and Hepa1-6 HCC cells to detect the expression levels of CFTR protein by western blotting. Analysis showed that the expression levels of CFTR in HepG2, MHCC97L and MHCC97H cells were lower than that in HL7702 cells, and the expression levels were negatively correlated with the metastatic potential of hepatoma cells (MHCC97H lower than MHCC97L lower than HepG2), while the expression levels of CFTR in Hepa1-6 cells were also lower than that in AML12 cells (Fig. 2E). Given CFTR’s critical role as a cAMP-regulated chloride channel, we hypothesized that its deficiency would disrupt cellular ion homeostasis. Subsequent intracellular chloride concentration measurements using MQAE fluorescence quenching confirmed significant Cl- accumulation in HCC cells compared to normal hepatocytes (Fig. 2F), consistent with compromised channel function.

### CFTR negatively regulates the proliferation and metastasis of HCC in vitro and in vivo

**Fig. 3:**
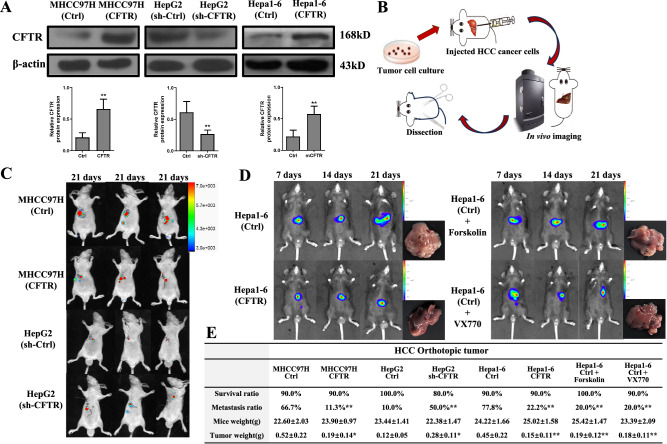
CFTR inhibited HCC proliferation and invasion in vivo. **A** Western Blot analysis of CFTR in overexpression MHCC97H cells, Hepa1-6 cells and interference of CFTR expression HepG2 cells (*n* = 3). **B** Flow chart of tumor bearing. **C** A representative imaging of orthotopic HCC tumors of MHCC97H Ctrl, MHCC97H CFTR, HepG2 sh-Ctrl, HepG2 sh-CFTR groups of BALB/c nude mice. **D** A representative imaging of orthotopic and Subcutaneous HCC tumors of Hepa1-6 Ctrl, Hepa1-6 CFTR, and Hepa1-6 Ctrl Forskolin groups of C57BL/6 J mice. **E** Survival, tumor formation, and tumor metastasis ratio of each group was calculated, and the weight of tumor and mice of each group was calculated (*n* = 10). Data are expressed as mean ± SEM. Statistical significance was concluded at *P < 0.05, **P < 0.01.

To verify the correlation between CFTR levels and the proliferation and metastasis of HCC, a HepG2 sh-CFTR cell line was constructed by using HepG2 cells with low metastatic potential and high levels of CFTR expression. In addition, MHCC97H/Hepa1-6 human CFTR (CFTR)/ mice CFTR (mCFTR) cell lines were constructed by using MHCC97H/Hepa1-6 cells with high metastatic potential and low levels of CFTR expression. After preliminary verification of CFTR overexpression and interference (Fig. 3A), the effects of CFTR levels on the proliferation of HCC cells were detected by MTT assays. Analysis showed that the proliferation rate of the HepG2 sh-CFTR group was significantly higher than that of the HepG2 sh-Ctrl group, while that of the MHCC97H CFTR group was significantly lower than that of the MHCC97H Ctrl group. The inhibitory effect of CFTR on HCC was time-dependent, and the cell proliferation effect was most significant at 72 h (Fig. S1A, B). PI staining and flow cytometry were used to detect the HCC cell cycle. Analysis showed that the overexpression of CFTR increased the proportion of MHCC97H cells in S phase and decreased the proportion of cells in G2 phase. After interfering with CFTR expression, the proportion of HepG2 cells in S phase decreased, while the proportion of cells in G2 phase increased significantly, thus promoting the transformation of MHCC97H cells from S phase to G2 phase; there was no significant effect on the proportion of cells in G1 phase (Fig. S1C, D). Transwell and scratch-wound healing recovery assays were used to detect the effects of CFTR on the invasion and migration of HCC cells. Analysis showed that the overexpression of CFTR significantly inhibited the invasion of MHCC97H cells, and CFTR interference significantly promoted the invasion of HepG2 cells. However, both overexpression and interference of CFTR had no significant effect on the migration of either MHCC97H or HepG2 cells (Fig. S1E, F).

In order to further verify the negative regulation of CFTR on the progression and metastasis of HCC, we used in vivo models of BALB/c nude mice transplanted with orthotopic HCC, C57BL/6 J mice transplanted with subcutaneous HCC, C57BL/6 J mice transplanted with orthotopic HCC (Fig. 3B). Firstly, four HCC cell lines (MHCC97H Ctrl, MHCC97H CFTR, HepG2 sh-Ctrl, HepG2 sh-CFTR) were transplanted into BALB/c nude mice in situ, while two HCC cell lines (Hepa1-6 Ctrl, Hepa1-6 CFTR) were transplanted into C57BL/6 J mice. After 3/4 weeks of tumor bearing, the liver and other tissues were removed from the experimental mice. Then, we calculated the growth rate and metastasis rate, and determined the tumor volume and tumor weight. Analysis showed that the overexpression of CFTR/ mCFTR in mice significantly reduced tumor weight, tumor volume and tumor metastasis rate in liver tissue when compared with the Ctrl group. Compared with the HepG2 sh-Ctrl group, the tumor weight, tumor volume and tumor metastasis rate in the liver tissue of mice in the HepG2 sh-CFTR group were significantly increased (Figs. 3C–E and S1). Forskolin and VX770, two commonly used CFTR activators (of channel function), demonstrated effects comparable to those observed with CFTR overexpression (Fig. 3D, E).

### CFTR is strongly correlated with the HCC immune microenvironment

**Fig. 4:**
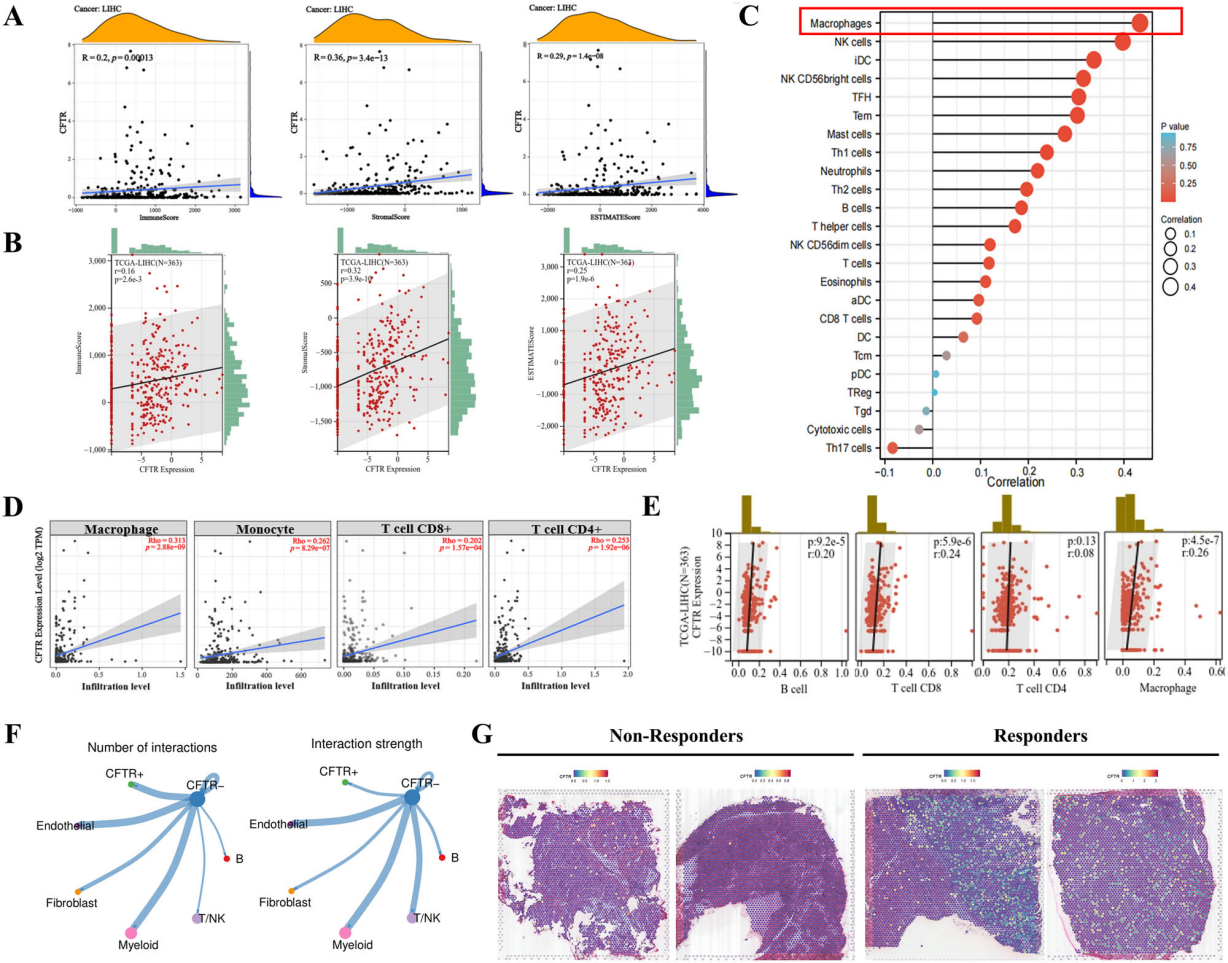
Correlation between CFTR and the tumor microenvironment in HCC. **A**, **B** CFTR expression has a negative correlation with immunescore, stromalscore, and ESTIMATEScore from TCGA and UCSC databases. **C** The relationship between CFTR expression and immune cells from TCGA databases. **D**, **E** The relationship between CFTR expression in several immune cells from TCGA and UCSC databases. **F** Single-cell analysis of intercellular communication between CFTR and immune cells. **G** The representative spatial transcriptomics results of CFTR in HCC.

In view of the significant inhibition effect of CFTR overexpression on the proliferation and metastasis of HCC in vivo, we speculated that other factors other than tumor cells might be involved in the mechanism of CFTR regulating the proliferation and metastasis of HCC. The immune infiltration scores showed that CFTR was significantly correlated with hepatocellular carcinoma (LIHC) (Fig. 4A, B). The scores for multiple immune cell infiltration were also evaluated; analysis showed that CFTR was significantly correlated with various types of immune cells in LIHC, especially macrophages and CD8 + T cells (Fig. 4C–E). Analysis of single-cell transcriptomic data revealed that CFTR is significantly correlated with multiple immune cell types (Fig. 4F). Furthermore, spatial transcriptomic data revealed that CFTR expression levels are negatively correlated with the response to PD-1 monoclonal antibody therapy: patients with high CFTR expression exhibited a higher response rate to immunotherapy, whereas those with low CFTR expression showed a poorer response (Fig. 4G).

According to the types of activation and different roles in tumor microenvironment, these cells can be divided into classical activated macrophages (M1) and alternatively activated macrophages (M2). These two polarization types of macrophages with opposite functions are closely related to the occurrence and metastasis of tumors. Therefore, we removed the tumor of BALB/c nude mice transplanted with orthotopic HCC (both MHCC97H-Ctrl and MHCC97H-CFTR), and flow cytometry was performed to detect the levels of M1/M2-polarized macrophages in each group. The results revealed that overexpression of CFTR and Forskolin intervention significantly promoted macrophage polarization toward the M1 phenotype while suppressing their polarization toward the M2 phenotype (Fig. S2A).

**Fig. 5:**
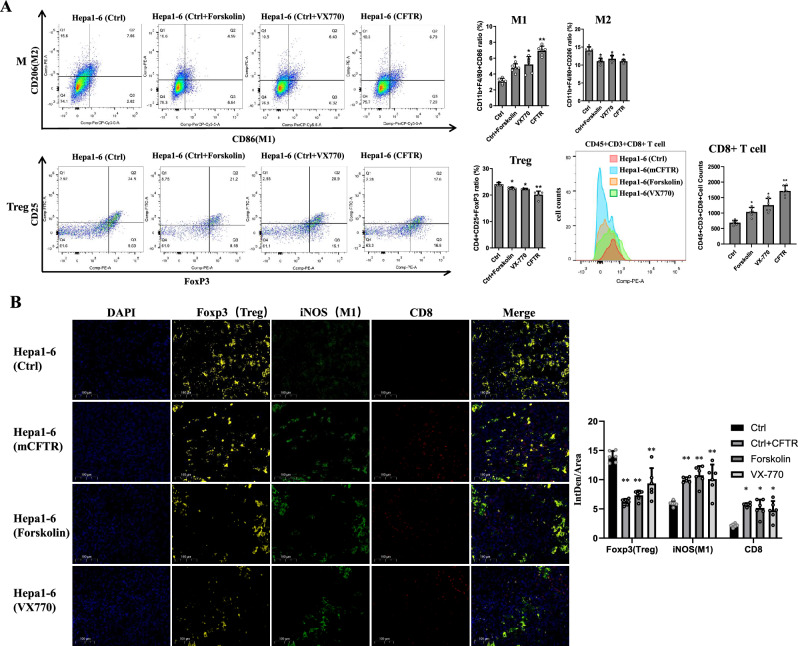
Effect of CFTR on the tumor microenvironment in C57BL/6 J mice. **A** Effect of CFTR overexpression and Forskolin/VX770 treatment on macrophage polarization, CD8 + T cells proliferation, and Tregs proliferation in tumor microenvironment of orthotopic hepatocellular carcinoma in C57BL/6 J mice (*n* = 6). **B** mIHC was employed to assess the effects of CFTR overexpression and Forskolin/VX770 treatment on the immune infiltration levels of T cells, M1 macrophages, and Tregs in hepatocellular carcinoma tissue of C57BL/6 J mice (*n* = 6). Data were expressed as mean ± SEM. Statistical significance was concluded at *P < 0.05, **P < 0.01.

However, due to the lack of a fully functional immune system in nude mice, Hepa1-6-Ctrl and Hepa1-6-CFTR cells were used to construct an orthotopic/subcutaneous HCC transplanted tumor model in C57BL/6 J mice. After 4 weeks of tumor bearing, we removed the tumor and the influences of CFTR on the polarization types of macrophages, and infiltrations of macrophages, CD8 + T cells and Treg cells were further detected by flow cytometry and mIHC. In flow cytometry analysis, CD11b and F4/80 double-positive cells were sorted from the tumor cell suspension and subjected to further analysis. The Q1 quadrant represented CD206-positive cells, indicative of M2 macrophages, while the Q3 quadrant represented CD86-positive cells, indicative of M1 macrophages. Cells positive for CD4, CD25, and FoxP3 were defined as Treg-positive cells, corresponding to the Q2 quadrant. The results showed that both over-expression of CFTR and Forskolin/VX770 intervention promoted the polarization to M1 and inhibited the polarization to M2, increase the infiltration levels of CD8 + T cells, and decreased the infiltration levels of Treg cells (Fig. 5A). In mIHC, the most representative marker proteins were selected for fluorescent labeling: FoxP3 for Tregs (yellow), iNOS for M1 macrophages (green), and CD8 for CD8 + T cells (red). The results showed that both over-expression of CFTR and Forskolin/VX770 intervention significantly enhanced the infiltration levels of M1 macrophages and CD8 + T cells into tumor cells, while reducing the infiltration of regulatory Treg cells (Fig. 5B). Consistent trends in the results were also observed in subcutaneous tumor-bearing mice (Fig. S2B–F).

Meanwhile, MHCC97H-Ctrl and MHCC97H-CFTR cells were also used to construct an orthotopic HCC transplanted tumor model in NOD-SCID mice with deep immunodeficiency; then, we re-infused macrophages (M)/ T cells/ M + T cells after one week. After 4 weeks of tumor bearing, we removed the liver tumor and other tissues from the experimental mice. Then, we calculated the rate of metastasis for each mouse and determined the tumor volume and tumor weight (Fig. S3A, B). Analysis showed that the overexpression of CFTR significantly reduced tumor weight and rate of tumor metastasis in liver tissue when compared with the MHCC97H Ctrl group. The tumor weight and tumor metastasis rate of mice reinfused with immune cells were significantly reduced. Of these, the tumor weight and rate of metastasis in mice reinfused with M and M + T cells were the lowest. Therefore, it is fully suggested that macrophages and CD8 + T cells are involved in CFTR-regulating HCC metastasis.

### CFTR modulated the RUNX1/NF-κB signaling axis in HCC cells to remodel the HCC immune microenvironment

**Fig. 6:**
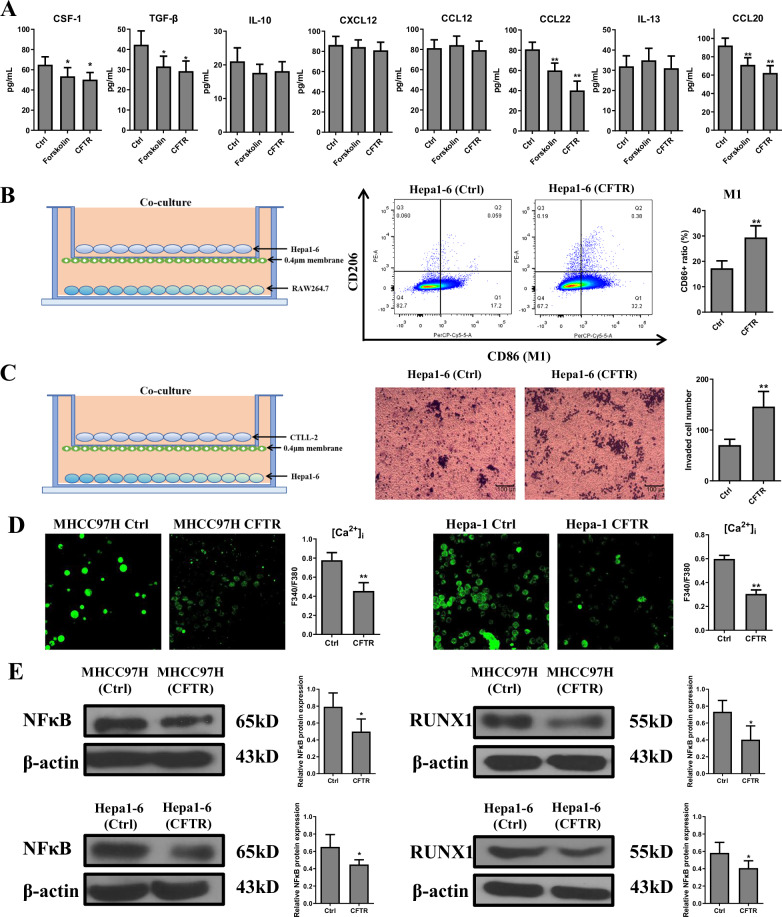
Verification of the potential mechanism of CFTR regulating HCC. **A** The release levels of multiple functional cytokines by Hepa1-6 cells (*n* = 3). **B** Pattern of co-culture of Hepa1-6 HCC cells with RAW264.7 cells and the effect of co-culture of Hepa1-6 cells overexpressing CFTR on RAW264.7 polarization (*n* = 3). **C** Pattern of co-culture of Hepa1-6 HCC cells with CTLL2 cells and the effect of co-culture of Hepa1-6 cells overexpressing CFTR on CTLL2 proliferation (*n* = 3). **D** Calcium (Ca^2+^) influx measurement was used to determine the influence of CFTR on intracellular calcium concentration (*n* = 3). **E** Western Blot analysis of NF-κB p65 and RUNX1 of each group in HCC cells. Representative images and the statistical analyses were shown. Data were expressed as mean ± SEM of at least three independent experiments. Statistical significance was concluded at *P < 0.05, **P < 0.01.

Macrophages polarize into functionally distinct subsets, including pro-inflammatory (M1) and anti-inflammatory (M2) phenotypes, whose dynamic balance is closely associated with tumor initiation and metastasis. Therefore, firstly we detected the release of several cytokines associated with macrophage polarization and CD8 + T cell infiltration in cell culture media of Hepa1-6 cells and found that both the overexpression of CFTR and administration of forskolin inhibited the release of CSF-1, TGF-β, CCL22, and CCL20 (Fig. 6A). Meanwhile, both the overexpression of CFTR and administration of forskolin inhibited the release of CSF-1, TGF-β, CCL22, and CCL20 in MHCC97H cells (Fig. S4A). These cytokines are closely associated with macrophage polarization, T-cell infiltration, and Treg recruitment. To further validate their expression patterns in HCC, we observed significant upregulation of these four cytokines in HCC tissues through TCGA database (Fig. S4B). These findings suggested their potential role as key factors promoting immune escape in HCC. Notably, enhanced CFTR function demonstrated a capacity to effectively downregulate its expression levels.

To further investigate the impact of CFTR overexpression in HCC cells on macrophage polarization and CD8 + T cell infiltration, Hepa1-6 cells were co-cultured with RAW264.7 macrophages or CTLL-2 T cells to assess their functional modulation. The results showed that CFTR overexpression was associated with a statistically significant increase in M1 macrophage polarization and CD8 + T cell migration (Fig. 6B, C).

Since CFTR is an ion channel protein, its abnormal expression may lead to abnormal function. Meanwhile, based on our previous findings, we initially identified that CFTR regulates HCC proliferation and metastasis due to its enhanced function. Therefore, we further examined the effect of CFTR on intracellular chloride and calcium concentration in MHCC97H cells and found that both CFTR overexpression and forskolin administration could decrease intracellular chloride and calcium concentrations (Fig. 6D).

Given that chloride ions and calcium ions are pivotal intracellular regulatory ions, abnormal concentrations of either can activate or suppress associated signaling pathways, thereby modulating the expression and release of relevant functional cytokines. To investigate the regulatory mechanisms of these two ions, we first examined the expression of NF-κB, a key transcription factor for specific cytokines. The results showed that both CFTR overexpression and forskolin administration reduced the expression level in nucleus. To further explore the underlying mechanism, predicted transcription factor sets (integration of hTFtarget, KnockTF, FIMO_JASPAR, PWMEnrich_JASPAR, ENCODE, CHEA, TRRUST, GTRD, and ChIP_Atlas) and chlorine functional protein sets (extracted from the UniProt database) were obtained, and the intersection of these gene sets was subsequently determined. By identifying key transcription factors associated with chloride-related functional proteins and cytokines, we ultimately identified RUNX1 as a critical protein regulated by chloride ions (Fig. S5A) (Table S1). Protein expression analysis revealed that CFTR overexpression and forskolin administration significantly reduced RUNX1 expression levels (Fig. 6E and S5B).

### CFTR significantly potentiates the inhibitory effect of the PD-L1 monoclonal antibody against HCC

Given that overexpression/gain-of-function of CFTR has been shown to partially remodel the immunosuppressive microenvironment in HCC, while concurrently promoting the proliferation and infiltration of CD8⁺ T cells and M1 macrophages, it is hypothesized that overexpression/gain-of-function of CFTR may potentiate the anti-HCC efficacy of PD-1/PD-L1 immune checkpoint blockade.

**Fig. 7:**
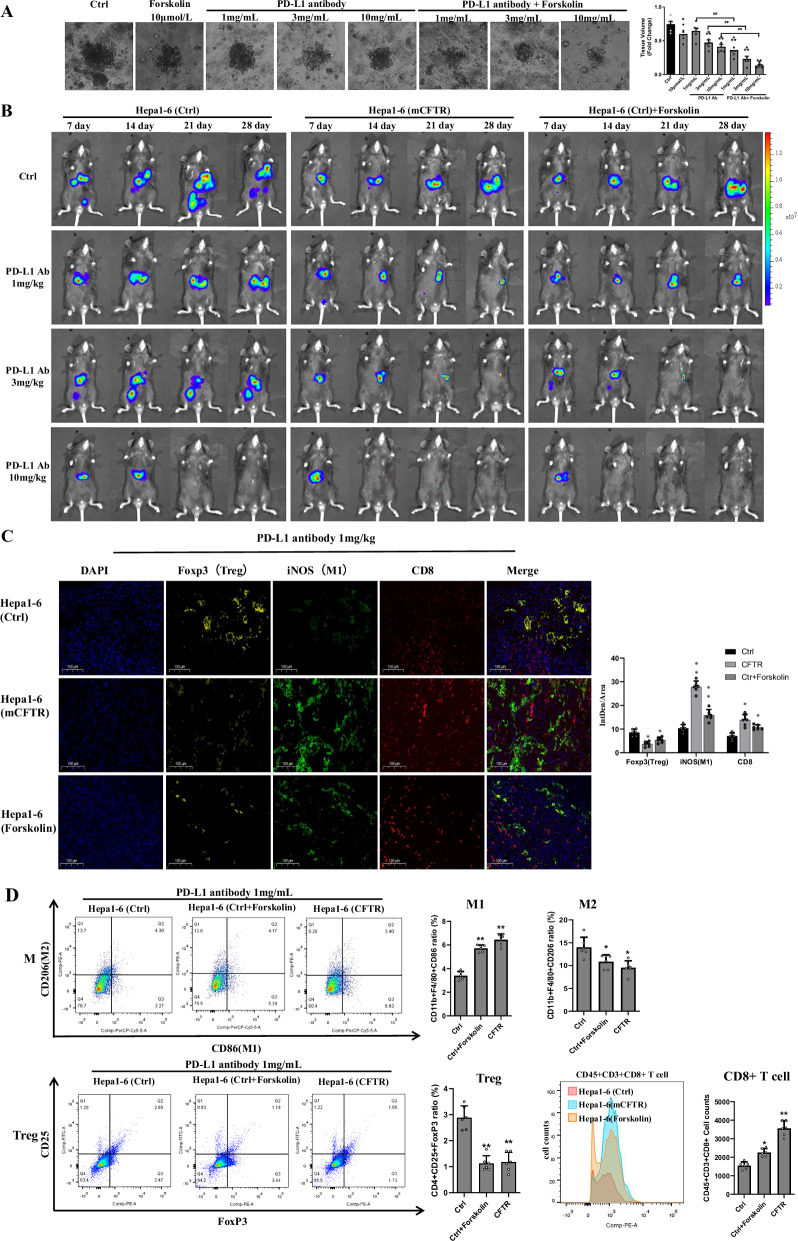
Validation of the potentiating effect of CFTR on PD-L1 antibody and the underlying mechanism in human HCC organoids and mice HCC models. **A** Validation of the potentiating effect of CFTR on PD-L1 antibody (*n* = 6). **B** A representative imaging of orthotopic HCC tumors of Hepa1-6 Ctrl, Hepa1-6 CFTR, and Hepa1-6 Ctrl Forskolin groups combined with PD-L1 antibody of C57BL/6 J mice. **C** mIHC was employed to assess the effects of each group on the immune infiltration levels of T cells, M1 macrophages, and Tregs in hepatocellular carcinoma tissue of C57BL/6 J mice (*n* = 6). **D** Effect of each group treatment on macrophage polarization, CD8 + T cells proliferation, and Tregs proliferation in tumor microenvironment of orthotopic hepatocellular carcinoma in C57BL/6 J mice (*n* = 6). Data were expressed as mean ± SEM. Statistical significance was concluded at *P < 0.05, **P < 0.01.

Therefore, human HCC tissue samples were used for organoid culture and drug effect detection. The results showed that Forskolin significantly inhibited the growth activity of human HCC organoids (Fig. 7A). Then, we established orthotopic and subcutaneous Hepa1-6 transplant tumor models in C57BL/6 J mice to evaluate the potential activity of CFTR to enhance the therapeutic effect of PD-L1 monoclonal antibody. The results showed that overexpression/gain-of-function of CFTR significantly inhibited tumor growth and metastasis in both orthotopic and subcutaneous models (Fig. 7B and S6). Subsequent mIHC and flow cytometric analyses of harvested orthotopic and subcutaneous HCC tissues at 4 weeks post-treatment revealed that, compared to PD-L1 mAb therapy, overexpression/gain-of-function of CFTR markedly augmented the infiltration levels of M1 macrophages and CD8⁺ T cells, while concomitantly suppressing the proliferation and infiltration of Tregs (Fig. 7C, D and S7A, B).

## Discussion

HCC cells are active in growth, strong in invasiveness, abundant in peripheral blood sinuses, and can readily invade capsules and blood vessels. These characteristics contribute to the metastasis of HCC and lead to high mortality rates. Treatment methods are constantly improving; however, the overall survival rate of patients has not been significantly improved. Therefore, we must identify new and more effective methods for the diagnosis and treatment of liver cancer.

An ion channel is a type of membrane protein that is responsible for regulating ion homeostasis inside and outside cells and has a complex biological function. Many studies have found that the abnormal expression of ion channel proteins is closely related to the occurrence and development of cancer [13, 14]. Furthermore, increasing evidence also suggests that dysregulation of ion channels may contribute to their survival and resistance to conventional therapies [16].

HCC is the main type of liver cancer, therefore, we investigated the expression levels of a certain channel protein (CFTR) during the early stages of HCC. Studies have shown that the detection rate of germline CFTR pathogenic variants (3.3%) was higher than the expected frequency (1.5%) based on population allele frequencies in an analysis of 2141 patients with solid tumors, particularly among those with skin and gastrointestinal cancers [17]. Additionally, CFTR expression has been found to be downregulated in various cancers, including HCC, and this reduction is associated with tumor progression. These findings collectively suggest that loss of CFTR function may increase cancer susceptibility through multiple mechanisms, whereas overexpression or gain-of-function of CFTR may exert tumor-suppressive effects by restoring its normal function.

We found that compared with normal adjacent tissues, the expression levels of CFTR, both mRNA and protein, in tissues from patients with HCC were significantly lower and that the expression levels of CFTR were positively correlated with the stage of HCC differentiation. Therefore, in the present study, we confirmed the expression levels of CFTR and their correlation with proliferation and metastasis in HCC using several clinical databases and HCC cells. Then, we constructed an MHCC97H cell line overexpressing CFTR and a HepG2 cell line that interferes with CFTR expression levels to further prove the correlation between CFTR levels and the proliferation and metastasis of HCC both in vitro and in vivo. Furthermore, we made an interesting discovery in that CFTR showed a more significant inhibitory effect on the proliferation and metastasis of HCC in vivo than that in vitro, thus suggesting some factors (other than internal factors of tumor cells) may be involved in the regulation of CFTR on HCC proliferation and metastasis.

The tumor microenvironment is one of the most important factors in regulating tumor metastasis. Therefore, we hypothesized that CFTR might interfere with immune escape by affecting the immune regulation of the microenvironment in HCC cells. Previous research showed that the loss of functionality caused by the down-regulation or mutation of some proteins in tumor tissues might be one of the main reasons for immune escape by tumor cells [18]. Therefore, to determine whether the negative regulation of CFTR is mediated by reducing its functional activity (channel activity), thus changing the concentration of related ions in cells, we used the CFTR activator forskolin [19, 20], a drug that can activate the CFTR channel activity without affecting its expression level. By performing in vitro and in vivo experiments, forskolin could inhibit the metastasis of HCC; the results were similar to the overexpression of CFTR. Therefore, it is possible that CFTR suppresses HCC metastasis through the enhanced function resulting from its overexpression, thereby inhibiting metastasis.

Homeostasis of the intracellular environment is closely related to the regulation of ion channels. The abnormal functionality caused by low CFTR expression will affect the intracellular concentration of chloride ions and then affect the intracellular calcium concentration [21, 22]. Chloride/calcium are directly involved in the proper synthesis of various proteins; meanwhile, abnormal intracellular chloride/ calcium concentrations will also lead to the abnormal expression of many proteins, including RUNX1 and NF-κB [23, 24]. Furthermore, the abnormal expression of RUNX1 and NF-κB will directly affect the expression of many functional cytokines.

In a tumor region, M2 macrophages stimulate angiogenesis by directly producing factors affecting tumor growth, such as chemokines (epidermal growth factor (EGF), CCL2 and CCL5) and cytokines such as VEGF, platelet-derived growth factor (PDGF) and macrophage colony-stimulating factor-1 (CSF-1). Furthermore, some cytokines, such as CSF-1, can further promote M2 polarization [25]. M2 macrophages can promote the secretion of proteases in the tumor microenvironment, such as urokinase plasminogen activator (uPA), cathepsin B and D, matrix metalloproteinase-2 (MMP2) and matrix metalloproteinase-9 (MMP9), thus promoting digestion of the tumor basement membrane and tumor escape [26, 27]. It can also promote cell movement and invasion through paracrine signaling pathways between tumor cells and M2 macrophages [28]. In addition, macrophages can also control Treg [25]. RUNX1 is a key transcription factor regulating the expression of multiple immunosuppressive cytokines [29]. It is closely associated with the expression and secretion of CCL20, CCL22, and TGF-β, which are critical factors for recruiting Tregs and M2 macrophages [30].

Since functional activation of CFTR modulates intracellular chloride and calcium homeostasis in HCC cells, thereby influencing the expression levels of RUNX1 and NF-κB. This cascade regulates the aforementioned cytokines, thus remodeling the HCC tumor microenvironment from an immunologically “cold” to a “hot” phenotype. Consequently, this transformation significantly enhances the therapeutic efficacy of PD-L1 immune checkpoint inhibitors against HCC.

To delineate whether the observed remodeling of the immune microenvironment stems from CFTR overexpression per se or from the functional restoration of its channel activity (resulting from overexpression), which subsequently modulates intracellular ion homeostasis, this study took the selective CFTR activator Forskolin as a control intervention. The results showed that forskolin also showed a significantly effects on tumor metastasis and proliferation in BALB/c nude, NOD-SCID, and C57BL/J6 mice in vivo and in human HCC organoid. This indicated that the functional enhancement caused by CFTR overexpression was the main reason for inhibiting HCC progression.

**Fig. 8:**
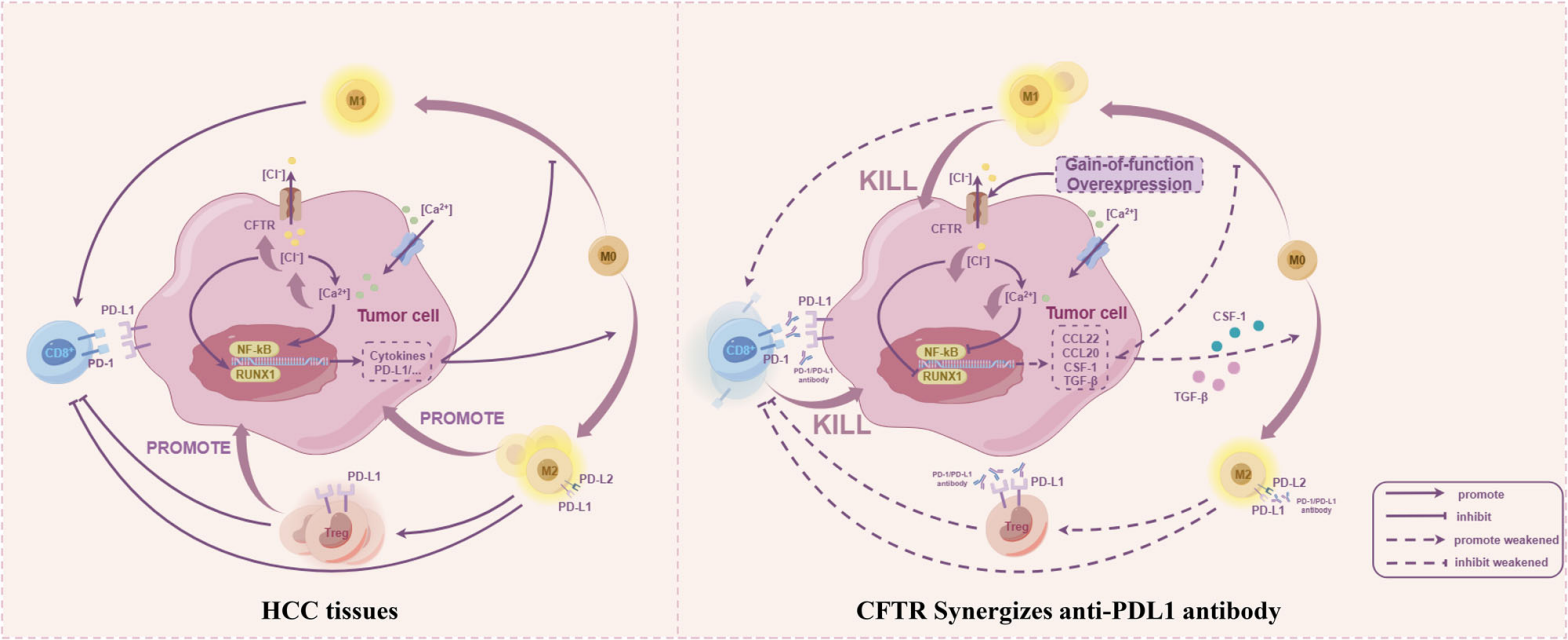
Potential pathway of CFTR-driven immune microenvironment reprogramming synergizes with PD-L1 antibody in hepatocellular carcinoma.

Collectively, our results suggested that the enhancement of CFTR function led to a reduction in intracellular chloride and calcium concentration, thus also reducing the expression of RUNX1 and NF-κB. This inhibited the release of CSF-1, CCL20, CCL22, and TGF-β, which promoted the transformation of M2 polarization into M1 polarization of macrophages, the increase of CD8 + T cells and the decrease of Treg cells, finally inhibited HCC proliferation and metastasis and enhanced the therapeutic efficacy of PD-L1 antibody against HCC (Fig. 8).

In conclusion, a new negative regulator of HCC proliferation and metastasis, CFTR, was identified. CFTR can regulate the ionic homeostasis and immune microenvironment of HCC, thus preventing the immune escape of liver cancer cells and enhancing the therapeutic efficacy of PD-L1 immune checkpoint inhibitors against HCC. These research results provide an important supplement to the existing theory of liver cancer occurrence and development from the perspective of immune anti-tumors and demonstrate the potential value of CFTR as an upstream biological target located in the liver cell membrane for the diagnosis and treatment of HCC. This study provides a valuable new target and a new mechanism of immune regulation to change the current situation of early metastasis, poor prognosis and refractory HCC.

## Supplementary information

Supplementary figures

Supplementary table

WB Figures

## Data Availability

Please contact the corresponding author for all data requests.
