# Supplementary material for: CFTR-driven immune microenvironment reprogramming synergizes with anti-PD-L1 antibody in hepatocellular carcinoma
Source: Cell Death Dis. 2026 Jun 10;17(1):731. doi: 10.1038/s41419-026-08907-7 (PMC13478323; doi:10.1038/s41419-026-08907-7)
Supplement: Supplementary file 1 [file 41419_2026_8907_MOESM1_ESM.docx]

**CFTR-Driven Immune Microenvironment Reprogramming Synergizes with anti-PD-L1 antibody in Hepatocellular Carcinoma**

**Yijia Xu^1,2,#^, Jianfang Sun^1,#^, Chongyang He^1^, Yipin Xie^1^, Yuying Lu^1^, Yujie Fan^1^, Liying Yang^1^, Mengying Zhang^1^, Xin Liu^1^, Lijuan Kong^1^, Yanfeng Liu^1^, Jinghai Zhang^1^, Yang Su^3,*^ and Mingyi Zhao^1,*^**

**Running title: CFTR Potentiates PD-L1 Blockade in HCC**

^1^ School of Life Sciences and Biopharmaceutical Science, Shenyang Pharmaceutical University, Shenyang, Liaoning, 110016, CN.

^2^ Department of General Surgery, Shengjing Hospital of China Medical University, Shenyang, Liaoning 110004, CN.

* To whom correspondence should be addressed:

*Prof. Mingyi Zhao, School of Life Sciences and Biopharmaceutical Science, Shenyang Pharmaceutical University, Shenyang, Liaoning, 110016, China, E-mail: [zhaomingyi@syphu.edu.cn](mailto:zhaomingyi@syphu.edu.cn).

*Prof. Yang Su, Department of General Surgery, Shengjing Hospital of China Medical University, Shenyang, Liaoning, 110000, China, E-mail: [suyang20131973@gmail.com](mailto:suyang20131973@gmail.com).

**^#^** These authors contributed equally to this work

**Figure S1**

**Figure S1** CFTR inhibited HCC proliferation and metastasis *in vitro* and *in vivo*.

A and B, MTT assays showed that overexpression of CFTR inhibited the growth of MHCC97H cells, and interference of CFTR expression promoted the growth of MHCC97H cells. E and F, MHCC97H cells with CFTR overexpression and HepG2 cells with interference of CFTR expression were subjected to Transwell and Scratch assays. Data are expressed as mean ± SEM of at least three independent experiments. Statistical significance was concluded at **P* < 0.05, ***P* < 0.01.

**Figure S2**

**Figure S2** Correlation between CFTR and the tumor microenvironment in HCC.

A, Effect of CFTR overexpression and Forskolin treatment on macrophage polarization of orthotopic hepatocellular carcinoma in BALB/C nude mice. B~E, Effect of CFTR overexpression and Forskolin treatment on macrophage polarization, CD8+ T cells proliferation, and Tregs proliferation in tumor microenvironment of subcutaneous hepatocellular carcinoma in C57BL/6J mice. F, mIHC was employed to assess the effects of CFTR overexpression and Forskolin treatment on the immune infiltration levels of T cells, M1 macrophages, and Tregs in subcutaneous hepatocellular carcinoma tissue of C57BL/6J mice. Data were expressed as mean ± SEM. Statistical significance was concluded at **P* < 0.05, ***P* < 0.01.

**Figure S3**

**Figure S3** Validation of the role of CFTR in regulating immune cells using a HCC model in NOD-SCID immunodeficient mice.

Data were expressed as mean ± SEM. Statistical significance was concluded at **P* < 0.05, ***P* < 0.01.

**Figure S4**

**Figure S4** Effects of CFTR on the release levels of multiple functional cytokines.

A, the release levels of multiple functional cytokines by MHCC97H cells (n=3). B, the expression level of multiple functional cytokines in HCC tissues of TCGA database.

**Figure S5**

**Figure S5** Verification of potential mechanism of CFTR regulating HCC.

A, prediction of key transcription factors governing functional cytokines modulated by chloride ions. B, Western Blot analysis of NF-κB p65 and RUNX1 of each group in HCC cells.

**Figure S6**

**Figure S6** Representative imaging of subcutaneous HCC tumors of Hepa1-6 Ctrl, Hepa1-6 CFTR, and Hepa1-6 Ctrl Forskolin groups combined with PD-L1 antibody of C57BL/6J mice.

**Figure S7**

**Figure S7** Validation of the potentiating effect of CFTR on PD-L1 antibody in mice subcutaneous HCC models. A, mIHC was employed to assess the effects of each group on the immune infiltration levels of T cells, M1 macrophages, and Tregs in hepatocellular carcinoma tissue of C57BL/6J mice (n=6). B, Effect of each group treatment on macrophage polarization, CD8+ T cells proliferation, and Tregs proliferation in tumor microenvironment of hepatocellular carcinoma in C57BL/6J mice (n=6). Data were expressed as mean ± SEM. Statistical significance was concluded at **P* < 0.05, ***P* < 0.01.
