# Supplementary material for: CFTR-driven immune microenvironment reprogramming synergizes with anti-PD-L1 antibody in hepatocellular carcinoma
Source: Cell Death Dis. 2026 Jun 10;17(1):731. doi: 10.1038/s41419-026-08907-7 (PMC13478323; doi:10.1038/s41419-026-08907-7)
Supplement: Supplementary file 2 [file 41419_2026_8907_MOESM3_ESM.docx]

https://pan.baidu.com/s/19g3M5wYcjDB1-a9SM7rObw

code: f8sr
